# A deep transfer learning approach for COVID-19 detection and exploring a sense of belonging with Diabetes

**DOI:** 10.3389/fpubh.2023.1308404

**Published:** 2023-11-06

**Authors:** Ijaz Ahmad, Arcangelo Merla, Farman Ali, Babar Shah, Ahmad Ali AlZubi, Mallak Ahmad AlZubi

**Affiliations:** ^1^Digital Transition, Innovation and Health Service, Leonardo da Vinci Telematic University, Chieti, Italy; ^2^Department of Engineering and Geology (INGEO) University "G. d’Annunzio" Chieti-Pescara, Pescara, Italy; ^3^Department of Computer Science and Engineering, School of Convergence, College of Computing and Informatics, Sungkyunkwan University, Seoul, Republic of Korea; ^4^College of Technological Innovation, Zayed University, Dubai, United Arab Emirates; ^5^Department of Computer Science, Community College, King Saud University, Riyadh, Saudi Arabia; ^6^Faculty of Medicine, Jordan University of Science and Technology, Irbid, Jordan

**Keywords:** COVID-19, deep learning, diabetes mellitus, chest x-ray, transfer learning, convolutional neural network, long-Covid

## Abstract

COVID-19 is an epidemic disease that results in death and significantly affects the older adult and those afflicted with chronic medical conditions. Diabetes medication and high blood glucose levels are significant predictors of COVID-19-related death or disease severity. Diabetic individuals, particularly those with preexisting comorbidities or geriatric patients, are at a higher risk of COVID-19 infection, including hospitalization, ICU admission, and death, than those without Diabetes. Everyone’s lives have been significantly changed due to the COVID-19 outbreak. Identifying patients infected with COVID-19 in a timely manner is critical to overcoming this challenge. The Real-Time Polymerase Chain Reaction (RT-PCR) diagnostic assay is currently the gold standard for COVID-19 detection. However, RT-PCR is a time-consuming and costly technique requiring a lab kit that is difficult to get in crises and epidemics. This work suggests the CIDICXR-Net50 model, a ResNet-50-based Transfer Learning (TL) method for COVID-19 detection via Chest X-ray (CXR) image classification. The presented model is developed by substituting the final ResNet-50 classifier layer with a new classification head. The model is trained on 3,923 chest X-ray images comprising a substantial dataset of 1,360 viral pneumonia, 1,363 normal, and 1,200 COVID-19 CXR images. The proposed model’s performance is evaluated in contrast to the results of six other innovative pre-trained models. The proposed CIDICXR-Net50 model attained 99.11% accuracy on the provided dataset while maintaining 99.15% precision and recall. This study also explores potential relationships between COVID-19 and Diabetes.

## 1. Introduction

COVID-19 is a severe and deadly disease caused by a newly discovered coronavirus. In late 2019, a strange sickness outbreak afflicted several people in Wuhan, China (1, 2). The precise reason for this widespread sickness outbreak was unknown, and the symptoms seemed strange. It has been determined that the virus has a unique coronavirus strain that was never found in humans before (1, 3). Coronaviruses can cause various respiratory diseases, ranging from moderate to severe. COVID-19 can be identified clinically by several symptoms related to the respiratory system, including pneumonia, cough, dyspnea, and fever. However, these signs are not exclusive to COVID-19 and can be seen in various pneumonia cases, which presents a challenge for medical professionals. Real-Time Polymerase Chain Reaction is one of the most accurate coronavirus testing techniques (RT-PCR), which has been authorized by the WHO (World Health Organization). In RT-PCR, the RNA sequence is converted into DNA through a process called reverse transcription, which is then amplified (4, 5). Global health, economy, and well-being were all affected by the rapid spread of the COVID-19 epidemic (6, 7). Detecting COVID-19 is critical for patient care and public health, as the pandemic can be avoided and effectively managed by isolating infected patients (8). It is critical to separate people infected with COVID-19; thus, early detection is a significant challenge for preventing the further spreading of the infection (9, 10).

Computer Imaging techniques in medical science can help control the spread of infection more effectively, treat infected individuals, and reduce the mortality rate (11–13). Imaging modalities such as computed tomography (CT) scans and chest X-rays (CXR) are essential for diagnosing pulmonary diseases (14, 15). In clinical practice, CXR and CT are frequently used to detect COVID-19. Even though CT has better sensitivity in COVID-19 detection, the CXR is a popular imaging modality because of its many advantages, including its low price, low level of radiation exposure, straightforward operation, and easy availability in hospitals (16, 17). Radiologists consistently face a clinical dilemma for COVID-19 detection during this pandemic (18). Rapid and precise COVID-19 identification is critical for avoiding and treating this pandemic disease through quarantine and medical treatment.

The lack of accessible testing kits makes it challenging to determine if the disease has spread as the number of reported cases rises. Deep Learning (DL)--based methodologies have progressed to the point where they can compete with the most advanced approaches in computer-aided diagnostics (19). In establishing computer-aided detection (CAD) systems, significant progress has been made using medical images and robust DL algorithms. These schemes are designed to automatically examine disease characteristics (20) using DL approaches to assist radiologists in making more accurate diagnostics. Recently, researchers applied DL algorithms to explore and assess CXR images for COVID-19 detection. COVID-19 detection and diagnosis methods using deep learning-based algorithms are precise and effective. The advantages of supervised DL algorithms in medical imaging tasks have been demonstrated in numerous applications (21). These DL algorithms need many data to create an accurate model. Unfortunately, access to such large volumes of labelled data is another significant problem for machine-learning approaches in the medical domain (22–24). A deep CNN model already pre-trained with variable layers can compensate for the lack of labelled data problems (24–26). Most initial layers in the pre-trained model are fixed to generic reserve aspects of natural images and train higher-level layers on medical images (27). This process of taking the pre-trained model of one problem and applying it to other related problems by retraining higher layers is called Transfer Learning (TL). Compared to standard DL algorithms, TL is simple, efficient, and has minimal training cost (27), thereby overcoming the problem of limited datasets.

Wang et al. described a Deep CNN-based system called Covid-Net for detecting COVID-19 instances in chest CXR images (28). This study helps doctors improve their transparency and screening when utilizing COVID-Net for computer-assisted screening by highlighting the significance of the main characteristics of COVID-19 cases. Apostolopoulos and Mpesiana suggested a TL-based technique for automatically recognizing COVID-19 using CXR images to assess how modern CNN designs classify data (29). The results suggest that extracting key COVID-19 disease characteristics by combining DL and X-ray imaging may be possible. Sethy et al. used CXR pictures to adapt the COVID-19 detection based on the extensive feature extraction and implementation of a Support Vector Machine (SVM) as a classifier (30). The study used thirteen different DCNN-based pre-trained models as feature extractors, providing each feature to the SVM classifier. ResNet50 model with SVM performs better than the other selected twelve classification models.

Hemdan et al. proposed a new COVIDX-Net system that uses seven DL pre-trained models to detect and analyze COVID-19 in two-dimensional CXR images (31). The findings of the proposed COVIDX-Net showed that the VGG19 and DenseNet201 models achieved the most significant performance scores among the other DL classifiers. Manokaran et al. employed a DenseNet201-based model for CXR image classification that was created by substituting a new network for the final classifier layer utilizing TL methods (32). Chakraborty et al. proposed a transfer learning approach based on VGG-19 pre-trained architecture to classify COVID-19, pneumonia, and healthy patients using CXR images (33). Ozturk et al. suggested a DarkCovidNet model for the CXR image classification (34). Binary and multi-class classifications are both supported by the model. An experienced radiologist carried out an analysis of how well the DarkCovidNet model worked. Jain et al. presented a two-stage process for classifying COVID-19 CXR images of persons with bacterial pneumonia, viral pneumonia, and healthy individuals (35).

Further analysis of the X-ray scans of viral pneumonia was performed to identify the presence of COVID-19. Their proposed DL model performs remarkably well in multi and binary classification phases. Vaid et al. created a model using the VGG19-based TL technique to improve its accuracy in detecting COVID-19 from CXR images (36). Pathak et al. proposed a ResNet50-based approach for building a COVID-19 CT image classification model (37). In terms of efficiency, their proposed classification model surpasses supervised learning approaches and achieves high accuracy. Karacan et al. proposed a binary and trinary classification system using CXR images (38). Their proposed model includes MobileNetV2, DenseNet121, InceptionResNetV2, and Xception. These models were integrated with ensemble learning methods to improve their proposed model’s performance further.

Narin et al. employed a Deep TL technique using chest radiographs to identify COVID-19 (39). The research used a technique known as five-fold cross-validation for three distinct binary classifications. Five models that had been pre-trained were applied to the three different datasets. According to their findings, The ResNet50 model offers the highest level of accuracy compared to the other four techniques included in the study. Using a precise weighted averaging ensemble model, Bhardwaj and Kaur attempt to detect COVID-19 and other pulmonary complications (40). Data augmentation strategies were implemented while training the four CNN models, DenseNet121, Xception, Inceptionv3, and InceptionResNetv2. The experiment’s binary classification accuracy was 98.33%, whereas, in the case of multi-class classification, they attained 92.36% accuracy.

The limitation of the previously mentioned research is that most of the studies used relatively limited CXR images, and others used very few COVID-19 radiographs. Some studies proposed binary classification models that cannot differentiate between bacterial and viral pneumonia. In this research, we proposed CIDICXR-Net50, a TL-based framework that uses a pre-trained model, ResNet50 architecture (41), for CXR image classification. Adopting the TL method reduces the impact of the problem of a restricted training dataset while providing us with the benefits of a shortened processing time, enhanced performance, and consistent results. The CIDICXR-Net50 model is built by substituting a new classification head for the last classifier layer in the ResNet50 model. The model is tested and trained using the dataset of 3,923 images, including 1,363 regular, 1,200 COVID-19, and 1,360 viral pneumonia Chest X-ray images, representing a sizeable dataset. The performance of the proposed CIDICXR-Net50 is assessed and compared with six other cutting-edge pre-trained models, including DenseNet-121, VGG-16, ResNet-101, VGG-19, InceptionV3, and MobileNetV2. The suggested CIDICXR-Net50 model achieved an accuracy of 99.11% on the provided dataset, with a 99.15% precision and recall rate.

- The current study offers a potential for cost-effective and swift diagnosis of Coronavirus disease using chest X-rays.
- This research presents a novel Deep Transfer Learning framework called CIDICXR-Net50, designed to aid radiologists in detecting COVID-19 from X-ray images with a high accuracy of 99.11%.
- In this study, we conducted a comprehensive performance evaluation of various deep learning architectures, offering insights into their accuracy in classifying COVID-19 based on an extensive X-ray image dataset.
- The proposed work facilitates collaborative efforts among interdisciplinary researchers to advance artificial intelligence methodologies within Computer-Aided Diagnosis (CAD) systems. To uncover potential linkages between COVID-19 and Diabetes Mellitus, thereby enhancing diagnostic precision and patient care strategies.

### 1.1. Relationship between COVID-19 and Diabetes

Diabetic patients, especially those with preexisting comorbidities or those in older age groups, have an elevated risk of COVID-19 infection (42). The trajectory of COVID-19 tends to be more severe for individuals with Diabetes, and they exhibit a markedly higher mortality rate (43). Through multivariable logistic regression analysis, Ciardullo et al. found that DM was an independent factor correlating with a rise in in-hospital mortality due to COVID-19 (44). Initial findings from China, subsequently supported by studies in the United States and Europe, revealed that the prevalence of Diabetes in individuals hospitalized with COVID-19 was as elevated as 20% (45–47). Emerging evidence indicates that Diabetes could potentially contribute as a risk factor for the occurrence of Post-Acute Sequelae of SARS-CoV-2 infection. After recuperating from the acute stage of COVID-19, certain individuals persistently suffer from symptoms over an extended duration, commonly known as “long COVID” or (PASC). Diabetic patients dealing with PASC may have difficulty controlling their blood sugar levels. There is still much to learn about the connection between COVID-19 and Diabetes, and research is underway. In order to provide Diabetes patients with the best care and outcomes possible throughout the pandemic, it is crucial to comprehend this link.

Once we confirm COVID-19 detection from the CXR image, then we can explore the relationship with Diabetes using some open datasets of electronic health records such as the National COVID Cohort Collaborative’s (N3C) repository, COVID-19 can disrupt glycemic control in people with Diabetes. Infection and the body’s immune response to the virus can lead to fluctuations in blood sugar levels, making it challenging for diabetic individuals to manage their condition effectively. Understanding the links between Diabetes and COVID-19 requires epidemiological, clinical, and molecular studies. Conditions that already exist, including a weakened immune response, viral replication, and persistent inflammation, are common contributors. These co-occurring conditions have also been linked to an amplified COVID-19 response. An impaired immune system is linked to poorly managed Diabetes. Individuals with Diabetes are at increased risk for severe complications from infections because their impaired immune systems cannot fight off the disease effectively. SARS-CoV-2, the virus responsible for COVID-19, may benefit from elevated blood glucose levels, speeding the course of the disease.

## 2. ResNet-50

**Figure 1:**
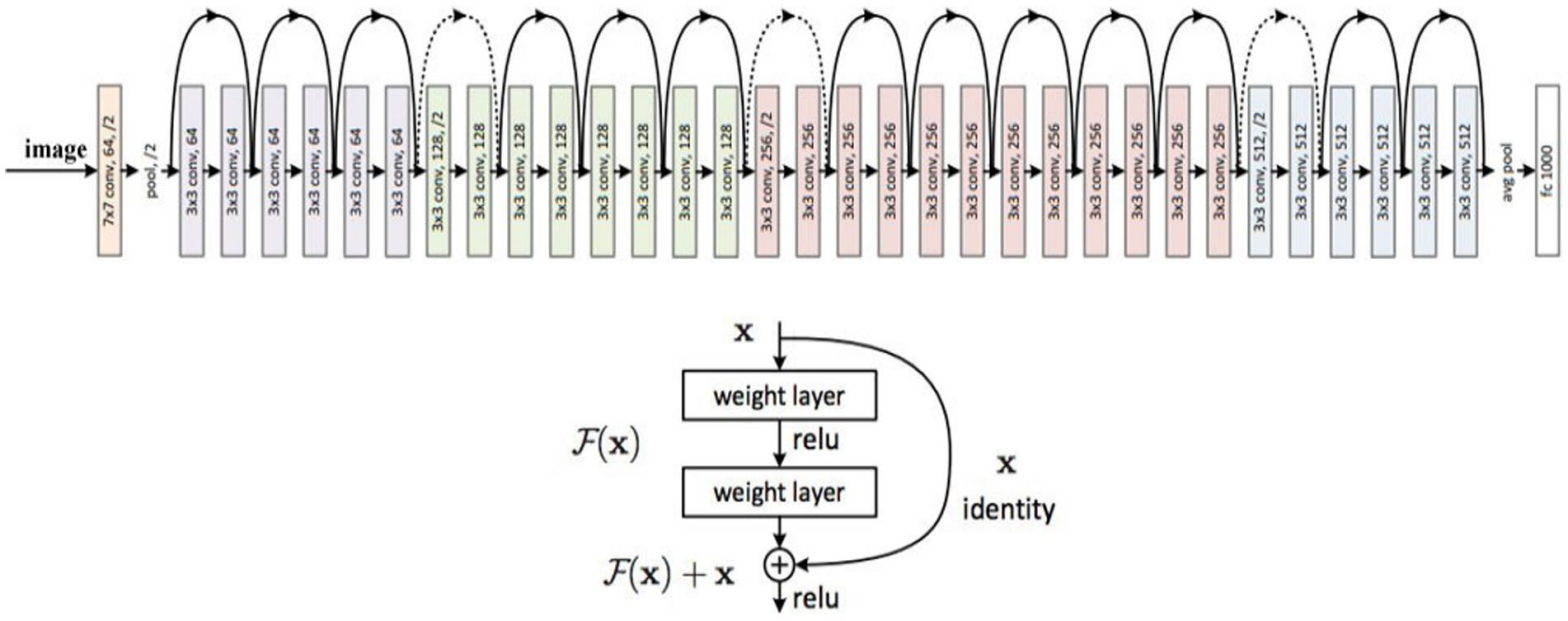
ResNet (Residual Network) architecture (41).

The ResNet-50 is a 50-layer Residual Neural Network (RNN) variant trained on images from the ImageNet database. The main reason for proposing the ResNet-50 model was to avoid the vanishing gradients problem while constructing a deep neural network. Different variants of the ResNet model are available with varying layers. However, the most common model is called ResNet-50, and it comprises 49 Convolutional layers and a Fully Connected layer. ResNet altered the structure of CNNs by introducing the residual learning technique to train deep neural networks. ResNet50 was nominated as the ImageNet Large Scale Visual Recognition (ILSVRC) Challenge winner in 2015. Figure 1 shows the design of the ResNet model.

ResNet is 20 and 8 times deeper than AlexNet11 and VGG42, respectively, offering more accuracy. The ResNet network with 50, 101, and 152 layers performs significantly better than the ResNet network with 18 and 34 layers. The design of the ResNet-50 network is made up of sequences of convolutional blocks that use average pooling. As the final classification layer, Softmax is employed. ResNet has established shortcut connections between different layers to enable communication between the different layers. As a result of the layers’ independence from parameters and data, non-residual functions can be characterized by them after a gated shortcut has been closed. In ResNet, shortcuts are never closed, but residual information is saved for good. Even as the search depth increases, it has lower computational complexity than VGG.

### 2.1. CIDICXR-Net50

**Figure 2:**
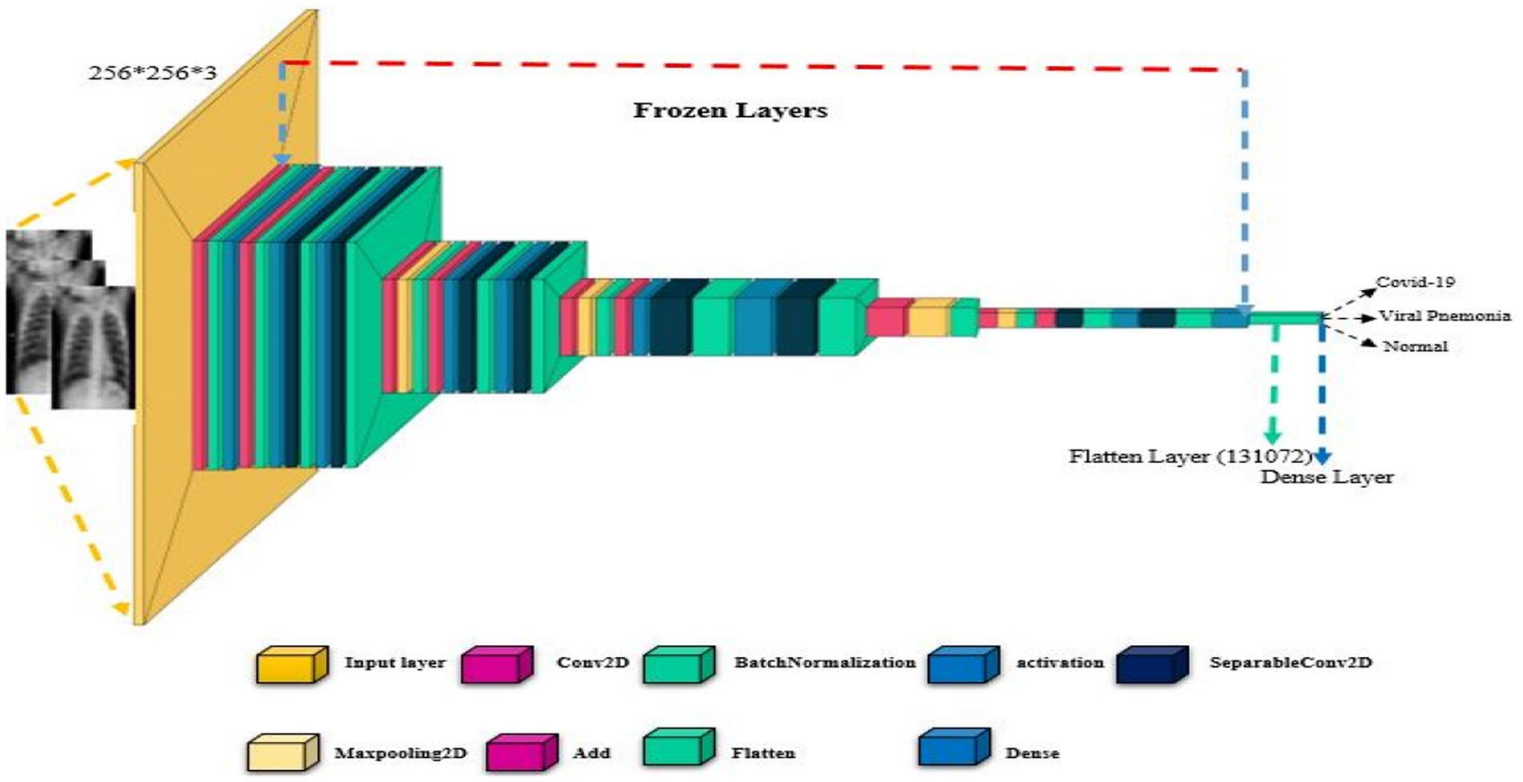
The architecture of the proposed CIDICXR-Net50.

The proposed CIDICXR-Net50 model is the modified version of the ResNet-50. In the CIDIXR-Net50 model, the network consists of one 7×7 convolutional layer, followed by three blocks of 1×1, 3×3, and 1×1 convolutional layers of size 56. Then, we have the same blocks of convolutional layers of sizes 28, 14, and 7. After that, instead of fully connected layers (as present in ResNet-50), we added flattened and dense layers. The flattened layer is used to transform the image into one dimension. The dense layer acts as a fully connected layer that uses Soft Max for multi-class classification (i.e., Normal, COVID-19, and Pneumonia). Figure 2 shows each layer of the original and proposed architectures sequentially. For both models, the first convolutional layer outputs a feature map of size 112 × 112 × 64 after applying 64 filters of size 7 × 7 × 3 over the input picture of size 224 × 224. The max-pooling layer processes the input feature map with a 3 × 3-pixel filter to create a 56 × 56 × 64 feature map. The initial 1×1 convolution layer is responsible for doing downsampling. However, in our proposed model CIDICXR-Net50, we use an input image of size 256 × 256 × 3 as it is the optimal image size. To effectively converge CNN models for training, hyper-parameters such as learning rate, optimization technique, dropout rate, and batch size must all be considered.

The proposed CIDICXR-Net50 model was trained using the Adam optimizer for 25 epochs, and the batch size was set to 32 with a learning rate 0.0001. A function called Early Stopping from the Keras library was also implemented. Model validation loss is tracked via this approach. If the model reaches its capacity and the validation loss continues to be constant, the best weights are kept, and the model is terminated. Activities at various levels of training were carried out using the ModelCheckPoint and EarlyStop callback methods.

### 2.2. Experimentation preliminaries

This section discusses the experimental tools, frameworks, techniques, dataset, preprocessing steps, hyperparameters of the proposed model, and performance assessment parameters used to conduct the experimentation. The proposed CIDICXR-Net50 model is implemented in Python using the Keras framework and TensorFlow 2.8.0 in the backend. The experiments were conducted in a Google Colab (Collaboratory) environment with an NVIDIA GPU, 12GB RAM, and 2.3GHz Intel Xeon Processors.

### 2.3. Dataset

**Figure 3:**
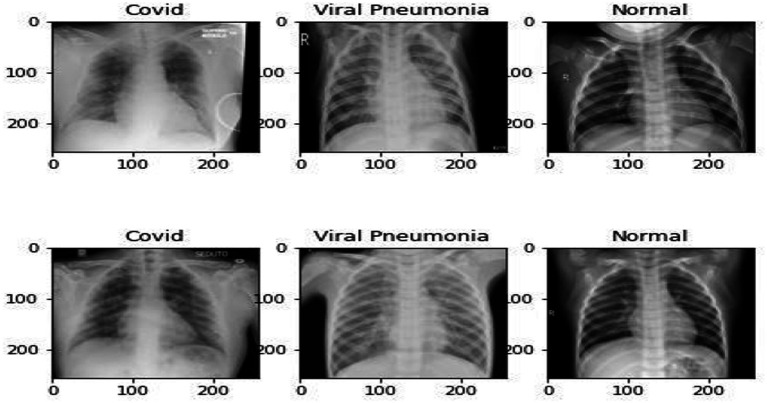
Preprocessed CXR images of Normal, Viral Pneumonia, and COVID-19.

The proposed model was trained and evaluated using version 3 of the publicly available dataset 43 of CXR images. The dataset included 3,923 total CXR images, 1,363 Normal, 1,200 COVID-19, and 1,360 Viral Pneumonia. The images included in the dataset are two-dimensional and have three channels. Figure 3 illustrates a CXR of infected lungs with Viral Pneumonia, COVID-19, and normal lung Chest X-ray images. The dataset is divided into training, validation, and testing. Details of the dataset and subsets are shown in Table 1.

**Table 1:** X-ray image dataset details.

Class	Training	Validation	Testing	Total CXR images
COVID-19	840	120	240	1,200
Normal	947	135	281	1,363
Viral pneumonia	956	135	269	1,360
Total CXR images	2,743	390	790	3,923

### 2.4. Data preprocessing

The dataset underwent preprocessing following the specifications of the suggested deep neural network model. Resizing and normalizing are the two essential procedures. Adjust the size of the CXR images to meet the specifications. The usual pre-trained models required fixed-size input images (such as 224 × 224, 227 × 227, 299 × 299), but the dataset contains images of varying sizes. As a result, all CXR images were resized to 256 × 256, and CXR images were normalized to [0,1] as an additional preprocessing step to meet the basic architecture’s requirements.

### 2.5. Hyper-parameters of the CIDICXR-Net50 model

The proposed CIDICXR-Net50 model was trained for 25 epochs using a 0.0001 learning rate and 32 batch size. Adam (adaptive moment estimation) optimizer is used to develop the classification model. Adam Optimizer, proposed by Kingma and Ba (48), is robust against noisy gradients and flexible enough to be used with different neural network architectures and tasks. Adam combines Momentum and RMSprop’s advantages to handle sparse gradients on noisy problems. The benefits of Adam Optimizer include Adaptive Learning Rates, Memory Efficiency, and Robust Variations. The Early Stopping function from the Keras library was implemented, monitoring the model’s validation loss. When the model reaches saturation but the validation loss remains the same, the best weights are preserved, and the model is halted. In this research, we used ModelCheckPoint and EarlyStop callback mechanisms. During training, the ModelCheckPoint mechanism ensures that the model is preserved with minimum data loss. If the network enters a state of inactivity (no learning), the EarlyStop method will be employed to interrupt the training of the system. As the validation loss monitoring parameter, the patience value was set to 10 initially. Rather than immediately halting the training when the measure stops increasing, “patience” inserts a buffer. It is the number of epochs you will wait for the metric to improve again. The value of patience is set to 10, which means that if validation loss does not decrease for ten consecutive epochs, the training process will be stopped. The Accuracy rate was employed as a performance metric in this case. The hyper-parameters used to train the CIDICXR-Net50 model are given in Table 2.

**Table 2:** Hyper-parameters of the proposed CIDICXR-Net50 model.

Hyper-parameters	Values
Optimizers	Adam
Learning rate	0.0001
EarlyStopping	patience = 10
Batch Size	32
Epochs	25
Callbacks	EarlyStop, ModelCheckPoint
Loss	Sparse Categorical Cross Entropy
Metrics	Accuracy

### 2.6. Performance assessment metrics

For performance evaluation and comparison of the proposed CIDICXR-Net50 and other models, we used Precision, Recall, F-measure (F1-Score), and Accuracy. These metrics are generated using the confusion matrix values, i.e., True Positive, True Negative, False Negative, and False Positive. Equations (1–4) illustrate the performance metrics mentioned above.


(1) $$\begin{array}{l} \mathrm{Accuracy}=\\ \frac{\mathrm{TruePositive}+\mathrm{TrueNegative}}{\mathrm{TruePositive}+\mathrm{FalsePositive}+\mathrm{TrueNegative}+\mathrm{FalseNegative}}\; \end{array}$$

(2) $$\mathrm{Precision}=\frac{\mathrm{TruePositive}}{\mathrm{TruePositive}+\mathrm{FalsePositive}}$$

(3) $$\mathrm{Recall}=\frac{\mathrm{TruePositive}}{\mathrm{TruePositive}+\mathrm{FalseNegative}}$$

(4) $$F1-\mathrm{Score}=\frac{2\times \mathrm{Recall}\times \mathrm{Precision}}{\mathrm{Recall}+\mathrm{Precision}}$$

## 3. Results

**Figure 4:**
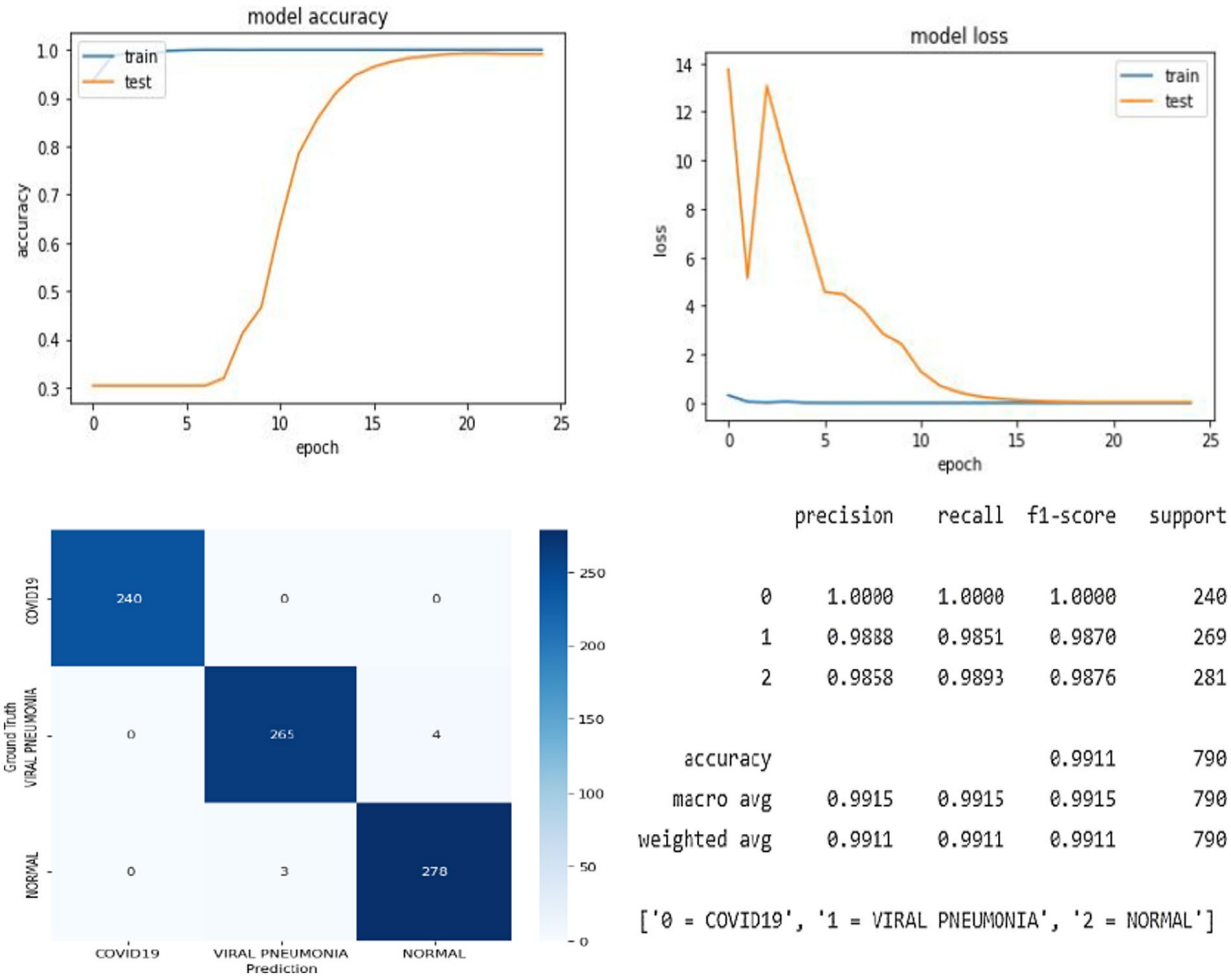
The overall result of the proposed CIDICXR-Net50 model.

In this research, we proposed the CIDICXR-Net50 (COVID-19 Infection Detection In Chest X-Ray) model to detect COVID-19 using CXR radiographic images. The CIDICXR-Net50 model uses the base structure of the pre-trained ResNet-50 model using a transfer learning approach. The study demonstrates that deep learning can facilitate the diagnosis process as our proposed automated diagnostic tool, the CIDICXR-Net50 model, achieved an accuracy score of 99.11% overall. The suggested model was validated using 790 CXR images, which included 240 COVID-19, 281 normal CXR images, and 269 viral pneumonia. Figure 4 illustrates the proposed model’s accuracy and loss.

**Figure 5:**
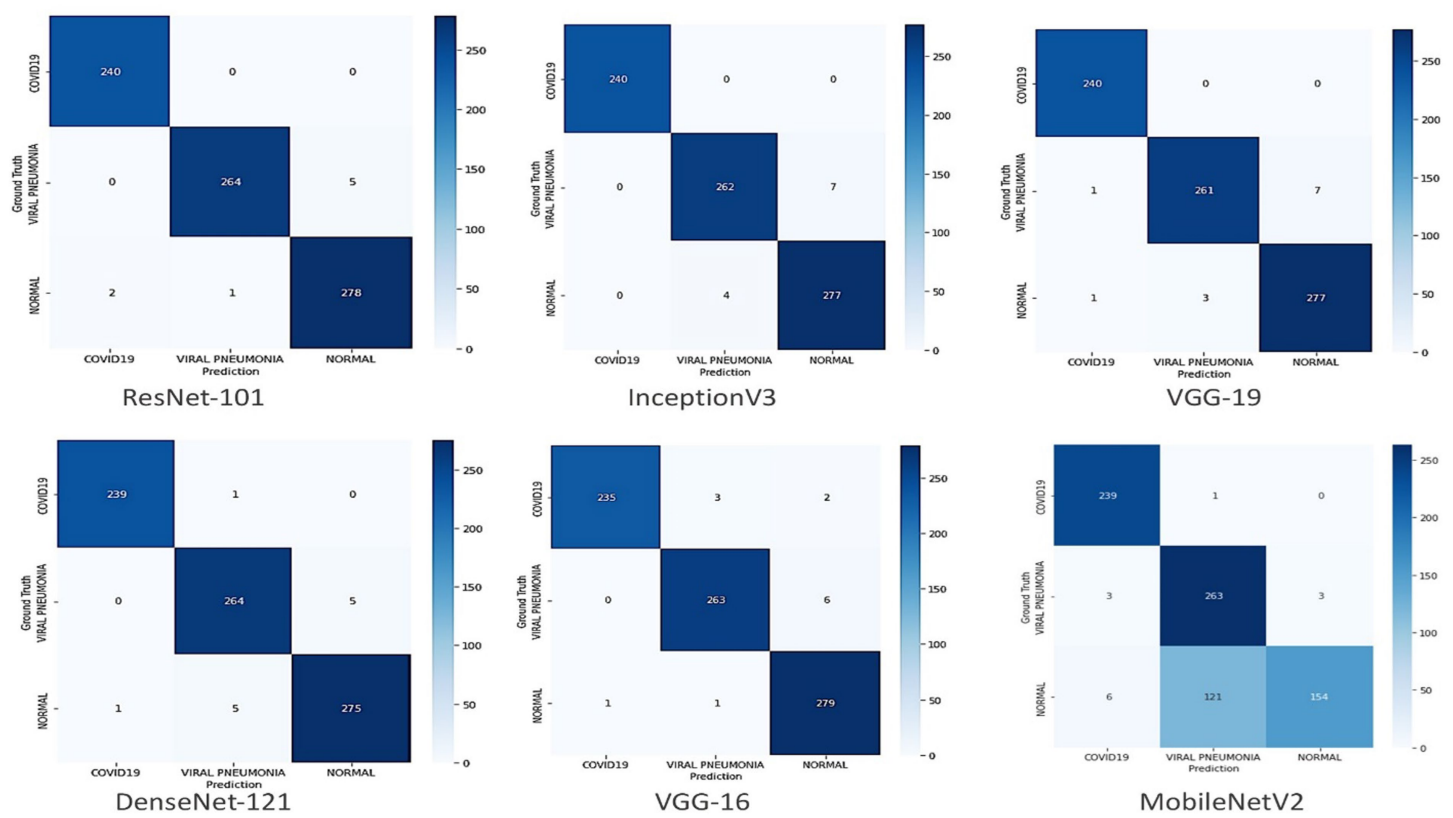
Confusion matrix for classification of COVID-19, normal and viral pneumonia using different deep learning models.

The trend graph shows the proposed model has no substantial overfitting or underfitting problems on the provided data. After 15 epochs, the performance curve for training and testing turns straight and progresses similarly. The proposed model accurately classified all 240 COVID-19 cases. Table 3 demonstrates each class’s Precision, Recall, and F1-Score of the CIDICXR-Net50. The performance of the CIDICXR-Net50 model was also compared with other well-known Deep Learning models, including VGG-16, VGG19, DenseNet-121, InceptionV3, ResNet-101, and MobilNetV2. All the performance comparison experiments are conducted on the same dataset and its subsets (i.e., Training, Validation testing) with default parameters. The results show that the proposed CIDICXR-Net50 model classified 99.11% of CXR images accurately compared to other selected models on the given data. ResNet-101 and InceptionV3 are second and third best, with 98.99 and 98.61% accuracy. The performance of these classification algorithms in terms of recall, precision, f1-score, and accuracy is illustrated in Table 4. The confusion matrix for classification of COVID-19, normal and viral pneumonia using Different Deep Learning models are shown in Figure 5.

**Table 3:** Precision, recall, and F1-Score of each class of the CIDICXR-Net50 model (proposed model).

CXR images class	F1-Score	Recall	Precision
Normal	0.99	0.99	0.99
COVID-19	1.00	1.00	1.00
Viral Pneumonia	0.99	0.99	0.99

**Table 4:** Performance of selected classification models for Chest X-ray classification.

Models	Accuracy %	F1-Score %	Precision %	Recall %
CIDICXR-Net50 (Proposed)	99.11	99.15	99.15	99.15
ResNet-101	98.99	99.01	99.01	99.02
InceptionV3	98.61	98.67	98.68	98.66
VGG-19	98.48	98.52	98.52	98.53
DenseNet-121	98.48	98.53	98.53	98.53
VGG-16	98.35	98.37	98.43	98.32
MobileNetV2	83.04	82.90	87.59	84.05

### 3.1. Discussion

This study aimed to construct a fully automated DL model called CIDICXR-Net50 to detect COVID-19 in chest X-ray images more accurately to classify COVID-19 CXR images from Viral Pneumonia and Normal CXR images. Previously, a hybrid technique was developed by Sethy et al. (30). Thirteen pre-trained DL models were used. An SVM classifier was trained using retrieved features from these models. ResNet-50 + SVM outperformed other classification models in ternary classification, with a sensitivity of 97.29% and an accuracy of 95.33%. The model was trained on 381 Chest X-ray images with an equal split across COVID-19, viral pneumonia, and normal. In contrast, the current CIDICXR-Net50 model accuracy and sensitivity are more significant by 3.78 and 1.82% on 11 times larger datasets.

Only 582 CT (Computed Tomography) scans have been used in the research. Multi-class classification accuracy of 99.11% and sensitivity of 99.15% are achieved by our proposed model, which was trained on a dataset five times larger (3923) than the one used by Pathak et al.DarkCovidNet automated model was suggested by Ozturk et al. (34) to detect COVID-19 in CXR. The model was developed for binary class (Normal and COVID) and multi-class classification (Normal, pneumonia, and COVID); it gained 87.02% for multi-class classification and 98.8% for binary classes. The CIDICXR-Net50 model reported 99.11% accuracy and 99.15% sensitivity on a vast dataset (Three times larger). A more notable increase of 12.11 to 13.62% was also observed in accuracy and sensitivity for trinary classification. Chowdhury et al. (49) created a binary-class and multi-class classification framework for automatically recognizing COVID-19 using a pre-trained DenseNet-201-based transfer learning technique.

The study employed 3,487 CXR images, and the networks were trained using binary and trinary classification methods. The binary and trinary classification accuracy were 99.70 and 97.94%, respectively, while the proposed CIDICXR-Net50 model yields a 99.11% accuracy and a 99.15% sensitivity. The suggested model is trained and evaluated using a comparatively massive number of COVID-19 CXR images (1,200 versus 423). Apostolopoulos & Mpesiana (29) examined the five pre-trained models Inception, VGG19, Xception, InceptionResNetV2, and MobileNetV2 for detecting COVID-19 in Chest X-ray images. A total of 1,442 CXR images, including 224 verified cases of COVID-19, were used in the investigation, representing just 15.53% of the entire dataset. The primary goal of this research was to separate COVID-19 from normal lungs CXR and Bacterial/Viral Pneumonia CXR. The sensitivity and accuracy rates for MobileNetV2 were the highest, at 98.6 and 94.72%. In comparison, the proposed CIDICXR-Net50 has a sensitivity of 99.15% and an accuracy of 99.11% on a relatively large dataset. Wang et al.24 developed a COVID-Net model for multi-class classification using an open-source CXR images dataset COVIDx. Accuracy and sensitivity for the CIDICXR-Net50 were 5.81 and 8.15% higher than reported (93.3 and 91%). Hemdan et al. (31) proposed a binary classification model COVIDX-Net using seven different pre-trained frameworks, including InceptionV3, InceptionResNetV2, Xception, VGG19, DenseNet201, ResNetV2, and MobileNetV2. The researchers trained and tested their model on 50 CXR pictures from 25 COVID-19 instances.

Jain et al. (35) developed a two-step process for detecting COVID-19 in CXR. In phase I, their model uses ResNet-50 to differentiate between bacterial and viral pneumonia in CXR images, including COVID-19. In multi-class classification, their model was 93.01% accurate. Phase II involved classifying COVID-19 CXR images from Viral Pneumonia using a pre-trained model based on ResNet-101. Experiments were performed on a dataset of 1,215 images that is publically accessible, and these experiments are supplemented further by data augmentation techniques. Their ResNet-101-based model achieved 97.22% accuracy. Manokaran et al. (32) suggested the DenseNet-201 base model, which detects COVID-19 CXR images with 94% accuracy. They used a dataset of 8,644 CXR images for experimentation, including 4,000 Normal, 4,000 Pneumonia, and only 644 COVID-19 cases. The results of their model outperformed other models by getting 92.19% accuracy. However, The key constraint was that their model was trained on a limited number of COVID-19 instances, and just 129 COVID-19 cases validated the conclusion.

In contrast, our proposed CIDICXR-Net50 model achieved 99.11% accuracy. Chakraborty et al. (33) proposed a VGG-19-based TL model to classify normal CXR, viral pneumonia CXR, and COVID-19 CXR. The accuracy of their model on the same dataset was 97.11%, whereas our proposed technique achieves 99.11% accuracy. The details of all previously mentioned research with their methods and accuracy are shown in Table 5.

**Table 5:** Comparative performance analysis of CIDICXR-NET50 against other leading techniques.

Model	Dataset	Methodology	Dataset size	Classification	Accuracy %
Sethy et al. (30)	CXR Images	TL ResNet50 + SVM	381	Multi-class	95.33
Pathak et al. (37)	CT Images	TL ResNet50	852	Binary-class	93.01
Ozturk et al. (34)	CXR Images	TL DarkCovidNet	1,127	Multi-class	87.02
Chowdhury et al. (49)	CXR Images	TL DenseNet-201	3,487	Binary-class	99.70
Chowdhury et al. (49)	CXR Images	TL DenseNet-201	3,487	Multi-class	97.94
Apostolopoulos & Mpesiana (29)	CXR Images	TL MobileNetV2	1,442	Multi-class	94.72
Wang et al. (28)	CXR Images	TL COVID-Net	13,975	Multi-class	93.3
Hemdan et al. (31)	CXR Images	TL COVIDX-Net	53	Binary-class	90
Jain et al. (Phase I) (35)	CXR Images	TL ResNet-50	1832	Multi-class	93
Jain et al. (Phase II) (35)	CXR Images	TL ResNet101	1832	Binary-class	97.78
Manokaran et al. (32)	CXR Images	TL DenseNet-201	8,644	Multi-class	92.19
Chakraborty et al. (33)	CXR Images	TL VGG-19	3,797	Multi-class	97.11
CIDICXR-Net50 (Proposed)	CXR Images	TL ResNet-50	3,923	Multi-class	99.11

## 4. Research limitations

Even though many medical imaging applications have achieved a good level of performance by utilizing deep learning models, many of these applications have failed clinical trials because of several issues, including a restricted training dataset, generalization, and overfitting. Training the CNN model on medical images instead of natural images (ImageNet) is recommended to obtain relevant medical characteristics. In this regard, a massive database of medical images is required to train the algorithm from scratch. Due to the recent disease outbreak and other factors, such as restrictions imposed by legal requirements that prohibit sharing patient CXR images, only a small amount of data is now available in open sources, which is inadequate to train the model from scratch. However, This research delves into the interaction between COVID-19 and Diabetes. However, due to the absence of openly accessible and pertinent electronic health records (EHR), this study did not present its own compiled findings that led to clinical practice.

## 5. Conclusion and future work

This study proposed CIDICXR-Net50, a deep ResNet50 base model, using a sizeable balanced dataset of CXR images and a TL technique to classify images of viral pneumonia, COVID-19, and standard CXR images. This study further delved into the intricate connection between Diabetes mellitus and its association with COVID-19. It was underscored that diabetic patients exhibit a heightened vulnerability to contracting COVID-19 and are more likely to develop post-acute sequelae of COVID-19 (PASC).To determine how well the suggested model performs compared to six other pre-trained models, including VGG-16, VGG19, DenseNet-121, InceptionV3, ResNet-101, and MobilNetV2. The proposed model outperformed the other selected models’ overall accuracy, efficiently separating patients diagnosed with COVID-19 from those diagnosed with normal or viral pneumonia. The results demonstrate that the proposed fully automated CIDICXR-Net50 model can detect COVID-19 infection with better accuracy. The CIDICXR-Net50 model proposed in this study can accurately detect COVID-19 from a dataset of ternary classes, another achievement of this research. The results of the experiments and assessments based on metrics show that the suggested model is suitable for use as a computer-aided diagnostics (CAD) system in hospitals and other medical facilities to diagnose COVID-19 disease in its early phases. This study supports the belief that deep learning algorithms have enormous potential for optimizing healthcare and improving diagnosis and treatment outcomes. The performance can be enhanced further in future work by increasing the dataset size. Collecting additional CXR images will increase the robustness and power of the proposed CIDICXR-Net50 model. To prevent overfitting issues and maximize generalizability, the developers of COVID-19 DL diagnostic models must train their models on vast and diverse datasets. Additionally, Because of the opaque nature of deep learning models, doctors may hesitate to rely on their results while making life-or-death decisions; therefore, Explainable Artificial Intelligence (XAI) techniques must be explored in the medical domain. Explanations are essential in the medical field, where every mistake might have severe consequences.

## Data availability statement

The original contributions presented in the study are included in the article/supplementary material, further inquiries can be directed to the corresponding author.

## Author contributions

IA: Conceptualization, Investigation, Methodology, Software, Supervision, Visualization, Writing – original draft, Writing – review & editing. AM: Conceptualization, Investigation, Methodology, Software, Supervision, Validation, Writing – original draft, Writing – review & editing. FA: Conceptualization, Investigation, Methodology, Software, Supervision, Writing – original draft, Writing – review & editing. BS: Conceptualization, Investigation, Methodology, Software, Validation, Visualization, Writing – original draft, Writing – review & editing. AA: Conceptualization, Investigation, Methodology, Software, Supervision, Validation, Visualization, Writing – original draft, Writing – review & editing. MA: Conceptualization, Investigation, Methodology, Software, Supervision, Validation, Visualization, Writing – original draft, Writing – review & editing.
